# Successful Management of Macula-Sparing Retinal Detachment Following Blunt Ocular Trauma Using Pneumatic Retinopexy

**DOI:** 10.7759/cureus.85709

**Published:** 2025-06-10

**Authors:** Ahmad Al-Saleh, Abdulaziz Al-Otabi, Yousef Al-Shammari, Mahmoud Alrabiah

**Affiliations:** 1 Ophthalmology, AlBahar Eye Center, Kuwait City, KWT; 2 Ophthalmology, Ministry of Health - Kuwait, Shuwaikh, KWT; 3 Ophthalmology, Albahar Eye Center, Kuwait City, KWT

**Keywords:** ocular injury, pan-retinal photocoagulation, retinal break, retinal detachment (rd), retinal detachment repair, retinal detachment surgery, retinal imaging, retinal injury, retinal trauma

## Abstract

In this study, we describe a case of traumatic macula-sparing retinal detachment secondary to blunt ocular trauma, involving a large superior retinal break, and evaluate the role of pneumatic retinopexy in its successful management.

A 54-year-old male sustained blunt ocular trauma resulting in a subtotal, macula-sparing retinal detachment with a large superior retinal break. He underwent pneumatic retinopexy with sulfur hexafluoride (SF₆) gas, followed by laser photocoagulation. Retinal reattachment was achieved within 24 hours. A secondary retinal break developed at follow-up and was managed successfully with focal laser. At one-month follow-up, best-corrected visual acuity was 20/40, and complete anatomical reattachment was maintained. This case highlights the expanding role of pneumatic retinopexy in selected trauma-induced retinal detachments, emphasizing the importance of patient selection, positioning compliance, and early detection of additional pathology.

## Introduction

Blunt ocular trauma is a well-recognized cause of rhegmatogenous retinal detachment (RD), often resulting from retinal breaks due to vitreoretinal traction. These cases are typically managed using pars plana vitrectomy or scleral buckling, especially in the presence of multiple breaks or poor visualization. However, pneumatic retinopexy (PR), when applied under strict anatomical conditions, is a viable, minimally invasive alternative with favorable outcomes [1].

Although PR has historically been reserved for primary, uncomplicated detachments, its indications are expanding. Several reports now support its use in more complex cases, including trauma-related detachments and giant retinal tears [1]. While our patient sustained direct blunt trauma, similar detachments have also been observed following acceleration-deceleration mechanisms [2].

Proper patient selection remains a key determinant of PR success, as outlined by Stewart and Chan [3]. The Pneumatic Retinopexy versus Vitrectomy for the Management of Primary Rhegmatogenous Retinal Detachment Outcomes Trial (PIVOT) randomized trial further demonstrated the non-inferiority of PR compared to vitrectomy in treating primary RD [4]. Additionally, a post hoc optical coherence tomography (OCT) analysis from the same trial indicated that PR may better preserve photoreceptor integrity than pars plana vitrectomy (PPV) in selected patients [5].

## Case presentation

A 54-year-old male presented to the ophthalmology clinic one day following blunt trauma to his left eye sustained from a football injury. He reported the sudden onset of floaters and a superior visual field defect. His medical and ocular histories were unremarkable.

On examination, best corrected visual acuity (BCVA) was 20/40 in both eyes, and intraocular pressure was within normal limits. Slit-lamp evaluation of the left eye (OS) revealed a clear cornea, a deep and quiet anterior chamber, and no evidence of hyphema or lens damage. Fundus examination demonstrated a subtotal retinal detachment associated with a large temporal retinal tear extending from 2 to 4 o’clock. The macula was spared.

**Figure 1:**
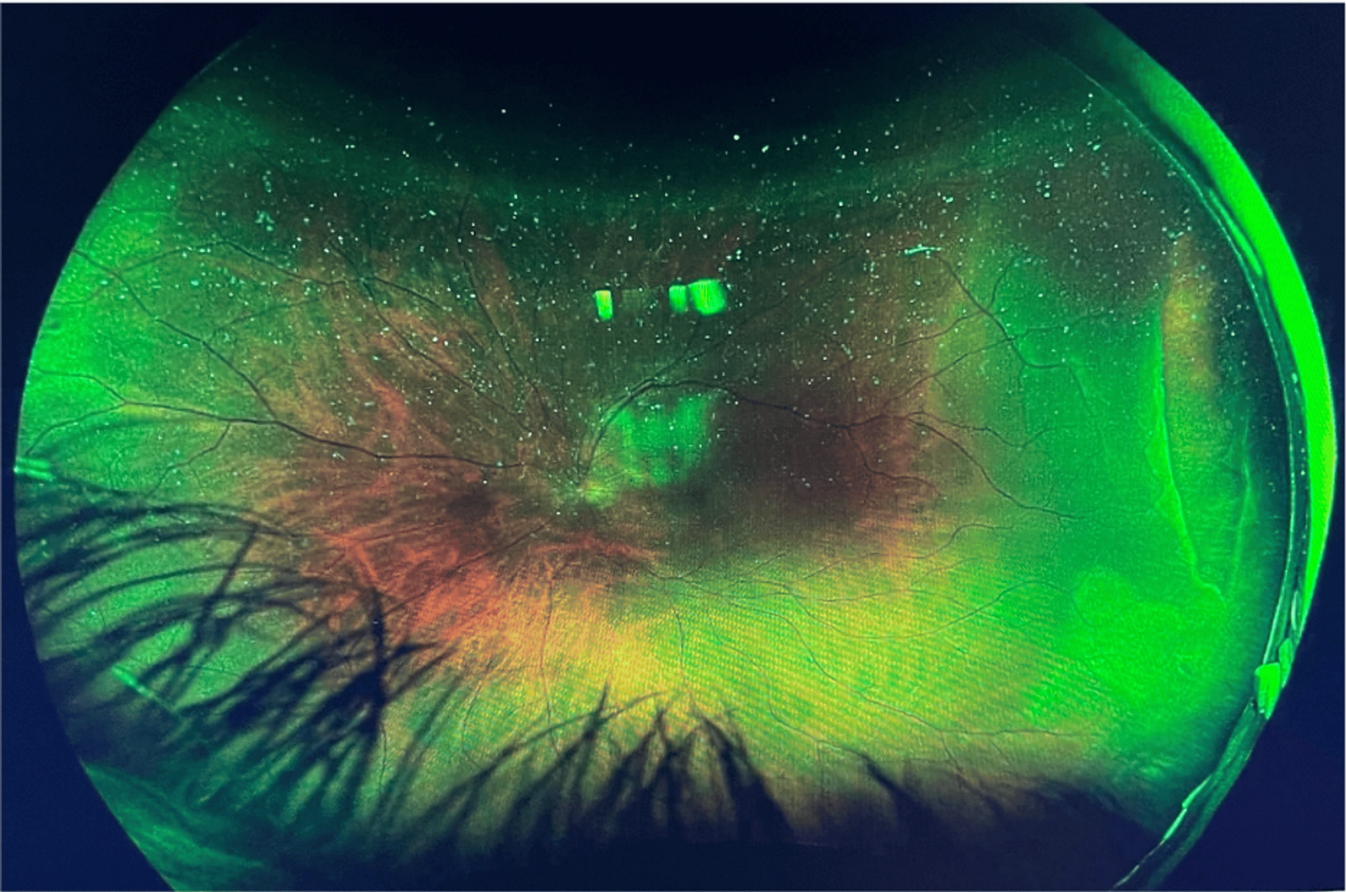
Wide-field Optos image showing a temporal tear from 2 to 4 o’clock with an associated temporal retinal detachment sparing the macula.

**Figure 2:**
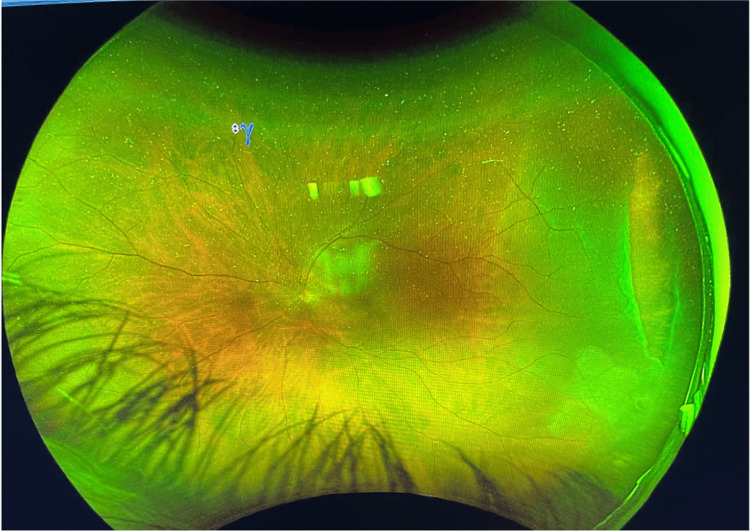
Another wide-field Optos image highlighting the same temporal tear and demonstrating macula sparing.

Wide-field Optos imaging confirmed the presence of a temporal retinal tear spanning from 2 to 4 o’clock with an associated temporal retinal detachment that did not involve the macula (Figures 1, 2).

**Figure 3:**
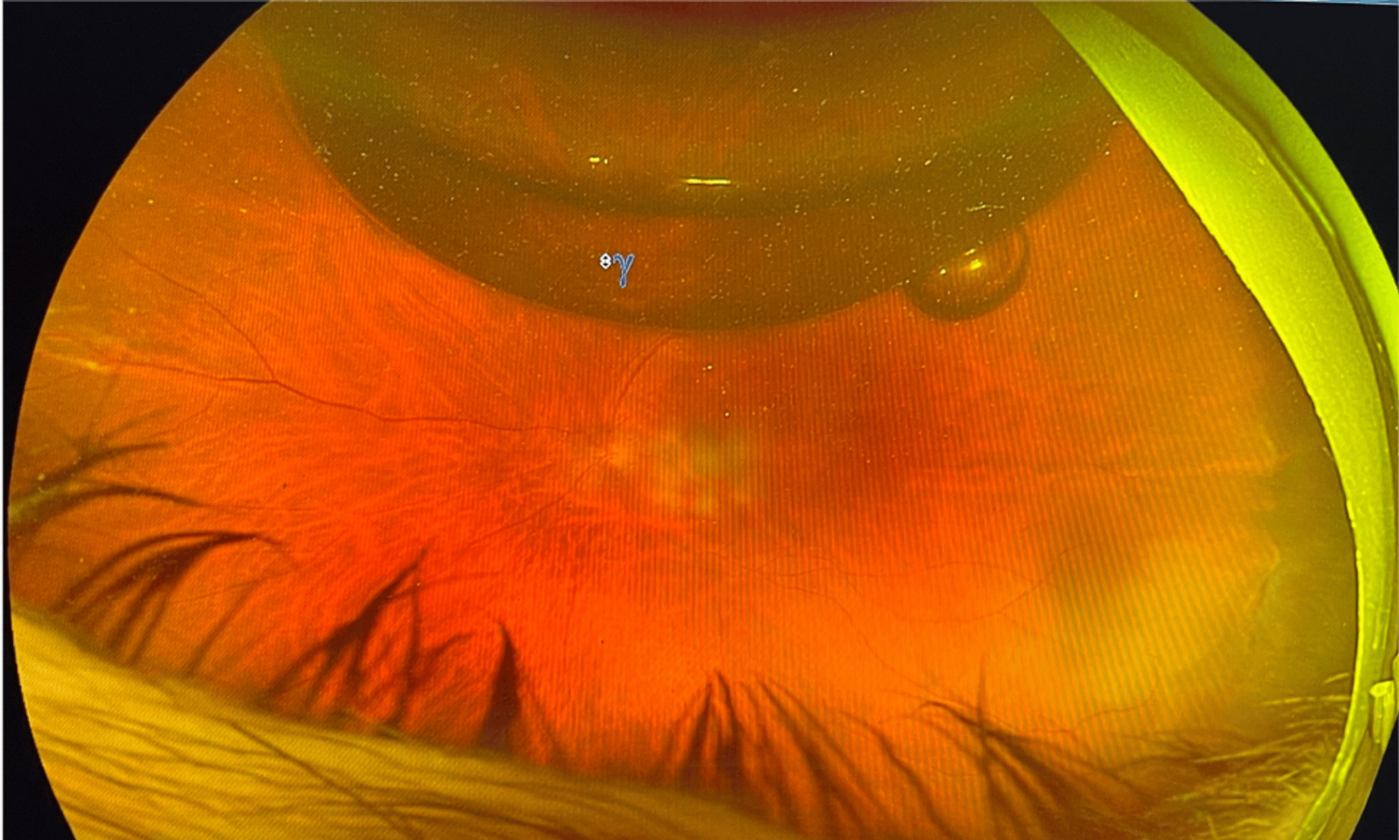
Wide field Optus image post pneumatic retinopexy.

The patient underwent pneumatic retinopexy under subconjunctival anesthesia. A 0.4 mL anterior chamber paracentesis was performed, followed by an intravitreal injection of 0.7 mL of pure sulfur hexafluoride (SF₆) gas. Prone positioning was maintained for 16 hours per day over seven days. Laser retinopexy was performed 24 hours after gas injection, following successful reattachment of the retina (Figure 3).

**Figure 4:**
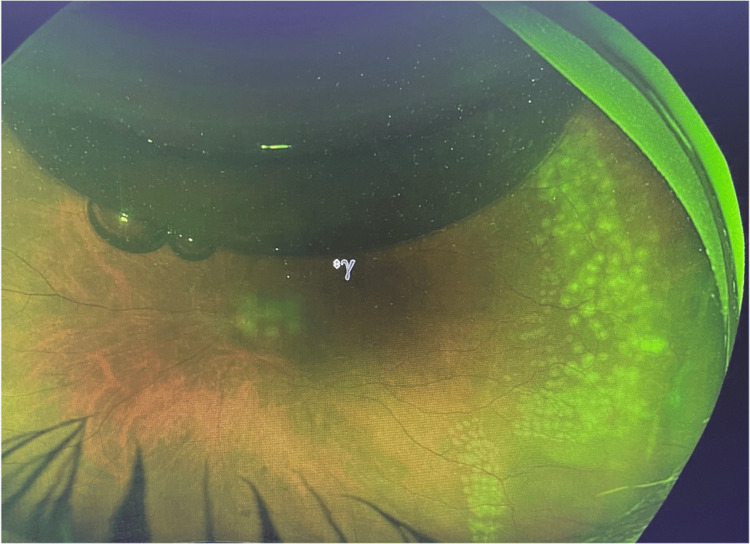
Wide field Optus image of the left eye post pneumatic and laser retinopexy for a temporal break spanning 2 to 4 o’clock. Retina is flat.

On postoperative day one, the retina was fully reattached. However, at the 10-day follow-up, a new retinal break was identified at the 7 o’clock position and was successfully treated with focal laser photocoagulation. Final imaging showed a flat retina and treated temporal break following pneumatic and laser retinopexy (Figure 4).

**Figure 5:**
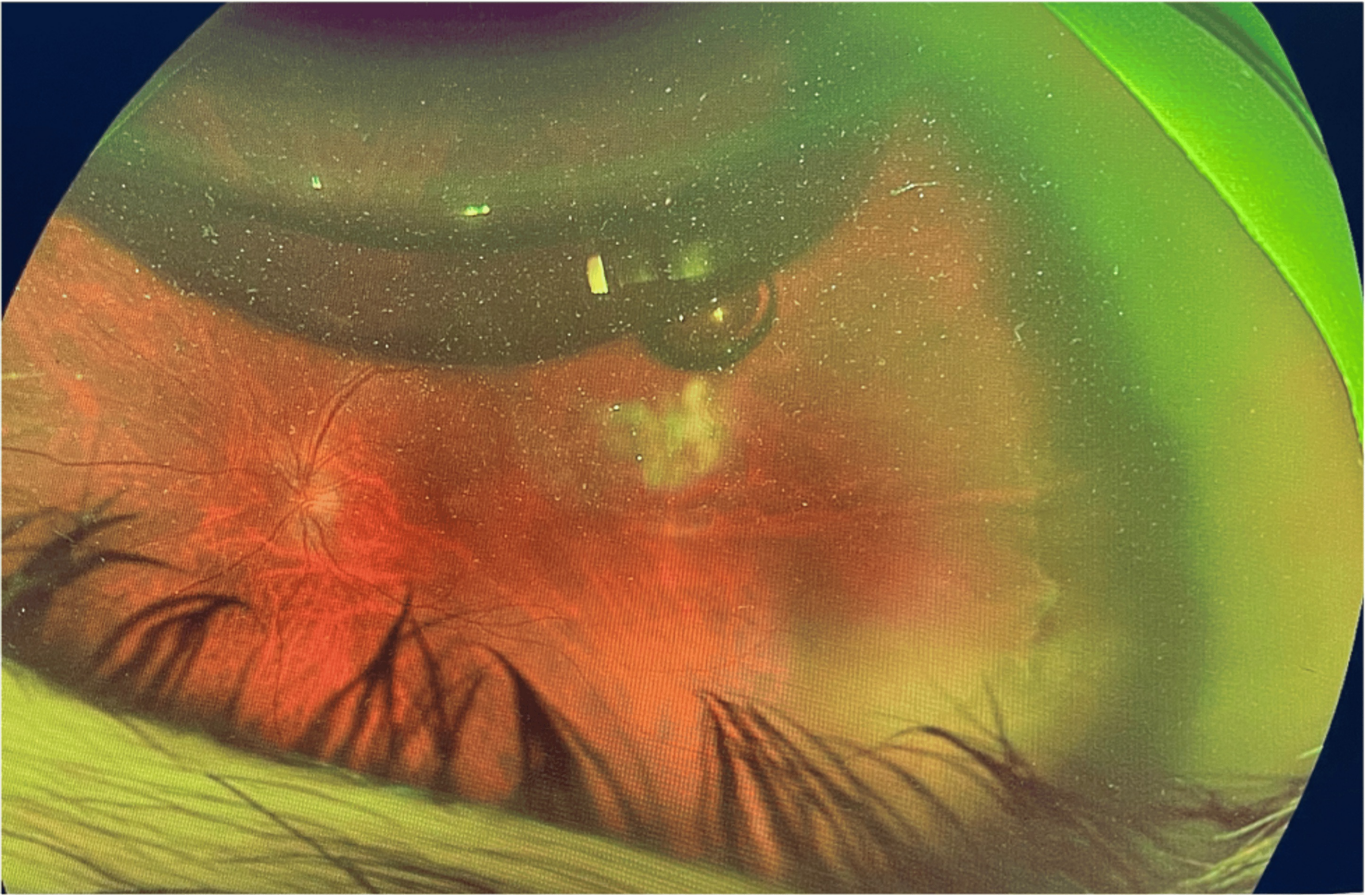
Wide field Optus image of the left eye post pneumatic retinopexy, temporal retinal break is present with a flat retina.

At the four-week follow-up, the retina remained attached, SF₆ was fully absorbed, and the BCVA was stable at 20/40. Final imaging showed a flat retina with treated breaks following pneumatic and laser retinopexy (Figure 5).

## Discussion

This case aligns with emerging evidence supporting the selective use of PR in trauma-related RDs. Although such cases have traditionally warranted PPV, favorable outcomes with PR have been reported in well-selected cases, particularly when the retinal break is single, located superiorly, and the macula remains attached [1,3]. Careful patient selection is essential to achieving optimal outcomes with PR. These findings are further supported by recent trials evaluating PR in traumatic and complex retinal detachments. The PIVOT trial and subsequent OCT-based sub-analyses have even demonstrated that PR may better preserve photoreceptor integrity compared to PPV in certain patients [4,5].

In our patient, prompt identification and treatment of a secondary retinal break likely prevented re-detachment, emphasizing the importance of meticulous follow-up and strict patient compliance. A well-structured postoperative monitoring plan is critical to detect new breaks early and apply timely interventions to maintain retinal attachment. This case illustrates that PR can be a safe and effective alternative to vitrectomy in trauma-related retinal detachments when appropriate case selection criteria are met. In particular, PR may offer advantages such as reduced invasiveness, lower cost, and potentially better preservation of photoreceptor structure and function in selected patients [1,3,5].

## Conclusions

Pneumatic retinopexy is an effective alternative to vitrectomy in selected cases of traumatic retinal detachment. This case supports its use in macula-on detachments with a single superior tear, provided that careful monitoring and timely intervention are ensured.
